# Experimental *in vivo* and *in vitro* treatment with a new histone deacetylase inhibitor belinostat inhibits the growth of pancreatic cancer

**DOI:** 10.1186/1471-2407-12-226

**Published:** 2012-06-08

**Authors:** Dmitriy I Dovzhanskiy, Stefanie M Arnold, Thilo Hackert, Ina Oehme, Olaf Witt, Klaus Felix, Nathalia Giese, Jens Werner

**Affiliations:** 1 Department of General Surgery, University of Heidelberg, Im Neuenheimer Feld 110, 69120, Heidelberg, Germany; 2 German Cancer Research Center (DKFZ), G340, Im Neuenheimer Feld 280, 69120, Heidelberg, Germany; 3 Department of Pediatric Oncology, University of Heidelberg Medical Center, Im Neuenheimer Feld 430, 69120, Heidelberg, Germany

**Keywords:** Pancreatic cancer, Histone deacetylase inhibitors, Belinostat, Mice

## Abstract

**Background:**

Treatment options for pancreatic ductal adenocarcinoma (PDAC) are limited. Histone deacetylase inhibitors are a new and promising drug family with strong anticancer activity. The aim of this study was to examine the efficacy of *in vitro* and *in vivo* treatment with the novel pan-HDAC inhibitor belinostat on the growth of human PDAC cells.

**Methods:**

The proliferation of tumour cell lines (T3M4, AsPC-1 and Panc-1) was determined using an MTT assay. Apoptosis was analysed using flow cytometry. Furthermore, p21^Cip1/Waf1^ and acetylated histone H4 (acH4) expression were confirmed by immunoblot analysis. The *in vivo* effect of belinostat was studied in a chimeric mouse model. Antitumoural activity was assessed by immunohistochemistry for Ki-67.

**Results:**

Treatment with belinostat resulted in significant *in vitro* and *in vivo* growth inhibition of PDAC cells. This was associated with a dose-dependent induction of tumour cell apoptosis. The apoptotic effect of gemcitabine was further enhanced by belinostat. Moreover, treatment with belinostat increased expression of the cell cycle regulator p21^Cip1/Waf1^ in Panc-1, and of acH4 in all cell lines tested. The reductions in xenograft tumour volumes were associated with inhibition of cell proliferation.

**Conclusion:**

Experimental treatment of human PDAC cells with belinostat is effective *in vitro* and *in vivo* and may enhance the efficacy of gemcitabine. A consecutive study of belinostat in pancreatic cancer patients alone, and in combination with gemcitabine, could further clarify these effects in the clinical setting.

## Background

Pancreatic ductal adenocarcinoma (PDAC) is a highly aggressive gastrointestinal tumour. It represents the fourth leading cause of cancer related deaths in both males and females in the western world [1]. Its resistance to most forms of therapy such as chemo-, radio-, immuno-, or targeted therapy contributes to the high mortality of this malignancy [2]. Curative resection is the only option to achieve long-term survival, but can be performed in only 15–20% of newly diagnosed pancreatic cancer patients [3].

Despite numerous findings that have contributed to the understanding [4] of PDAC pathogenesis, the reasons for its aggressive biological behaviour remain unclear. Therefore, new drugs for pancreatic adenocarcinoma are urgently needed. One of the most encouraging fields in modern oncological research is the investigation of epigenetics. Histone deacetylase inhibitors (HDACi) are typical representatives of this field of research, and may serve as promising agents with strong anticancer activity. The mechanism of HDACi activity is remodelling of chromatin through a modification of histone molecules, with consecutive initiation of cell differentiation, cell-cycle arrest and induction of apoptosis. Additionally, HDACi might affect neoplastic growth by regulating host immune responses and tumour vasculature [5]. Clinical studies have shown that HDACi can be administered safely in humans, and that treatment with these agents seems to be beneficial for some types of cancers [6,7].

A new hydroxamate type HDACi, belinostat (previously called PXD101), has been shown to be a potent anti-tumour agent with sub- to low micromolar IC50 potency in several tumour cell lines. In animal studies, belinostat specifically inhibited tumour growth at non-toxic concentrations [8,9]. Moreover, this compound has recently been evaluated in clinical phase I and II studies in both haematological and solid malignancies [10,11]. However, its efficacy in pancreatic cancer is unknown. Therefore, the aim of this study was to examine the efficacy of the novel pan-HDAC inhibitor belinostat on the growth of human PDAC cells *in vitro* and *in vivo*.

## Methods

### Cell culture and treatment

PDAC cell lines Panc-1 and AsPC-1 were obtained from the American Type Culture Collection (Manassas, VA, USA). T3M4 was a gift from R.S. Metzgar (Duke University, NC, USA). Cells were cultured in RPMI 1640 medium supplemented with 10% foetal bovine serum (FBS), 100 U/ml penicillin, and 100 μg/ml streptomycin (Invitrogen GmbH, Karlsruhe, Germany) at 37 °C in a humidified 5% CO_2_ atmosphere. When cells were subconfluent, medium was changed on both untreated and treated cells. Cells were treated with defined concentrations of a belinostat (Topotarget, København, Denmark) stock dissolved in DMSO. Control cells were grown following a similar protocol in medium supplemented with equivalent volumes of PBS.

### MTT assay for cell proliferation

A 3-(4,5-dimethylthiazol-2-yl)-2.5-diphenyltetrazolium-bromide (MTT) (Sigma-Aldrich Chemie, Steinheim, Germany) assay was used to assess cell proliferation reflected by metabolic activity of the cells. The cells were seeded in 96-well plates at a density of 5000 cells/well in 250 μl of complete medium. After the cells became adherent (in 24 h), they were exposed to belinostat (25nM, 50nM, 100nM, 300nM, 500nM, 800nM, or 1000nM) for 48 h. Cells exposed to PBS served as controls. After the indicated times, 10 μl of MTT (5 mg/ml, dissolved in PBS, pH 7.4) were added to each well and incubated for 4 h. After incubation, the culture medium was aspirated and the plates were dried by inversion for about 15 min. Subsequently, the formazan products were solubilised with acidic isopropanol (100 μl for each well) and the optical density was measured at λ = 570 nm with a Multiscan EX plate reader (Thermo Electron). All assays were performed in triplicate. Proliferation in the control group was set as 100%.

### Immunoblotting

After 24 h treatment with Belinostat (100 nM for T3M4 and ASPC or 300 nM for Panc-1), cells were washed twice with ice cold PBS before lysis with SDS lysis buffer (2% w/w SDS, 1 mM DTT, 10% v/v Glycerol, 62.5 mM Tris–HCl pH6.8, 0.05% w/v Bromphenolblue). Protein concentration was determined by BCA protein assay (Pierce Chemical Co., Rockford, IL, USA). Cell lysates (30 μg protein/lane) were separated on a 15% SDS/polyacrylamide gel and electroblotted on PVDF membrane (Whatman, Maidstone, UK). Membranes were then incubated in blocking solution (5% milk in 20 mM Tris–HCl, 150 mM NaCl, 0.1% Tween-20), followed by overnight incubation at 4 °C with anti acH4, CHIP-Grade antibody (Upstate Biotechnology, Billerica, USA) at a dilution of 1:75,000, or rabbit polyclonal anti- p21^Cip1/Waf1^ (Abcam, Cambridge, UK) at a 1:200 dilution. The membranes were then washed in TTBS and incubated with secondary antibodies: POD conjugated donkey anti-rabbit (Promega, Mannheim, Germany) at 1:150,000 dilution (blot for acH4) or POD conjugated goat anti-rabbit IgG (GE Healthcare Limited, Buckinghamshire, UK) at 1:2000 dilution (blot for p21^Cip1/Waf1^).

Antibody detection was performed using an enhanced chemiluminescence reaction (Amersham Bioscience). Equal lane loading was confirmed using anti-actin (Clone AC-15) antibody (Sigma-Aldrich Chemie). The actin signal obtained after incubation with anti-actin antibody on the same membrane was used as an internal control, in addition to loading all lanes with the same amount (30 μg) of protein. All assays were performed in triplicate. For the semiquantitative analysis of the immunoblots, densitometry using the ImageJ program was carried out, and the signal intensity of acH4 or p21^Cip1/Waf1^ expression was normalised to its corresponding signal intensity of actin.

### Induction and analysis of cell death by flow cytometry

Belinostat was diluted in phosphate-buffered saline (PBS) to a final concentration ranging from 100 to 1000 nM, according to the concentrations indicated in each experiment. Gemcitabine (Synchem OHG, Felsberg, Altenburg, Germany) was applied at a final concentration of 0.01 mM in PBS. All cells were treated and cultivated under the same conditions (37 °C with 5% CO2 in RPMI 1640), and exposed to the drugs 24 h before the experiments.

The viability of PDAC cells after exposure to belinostat and/or gemcitabine was analysed using annexin V/propidium iodide (PI)-staining (Annexin V-FITC Apoptosis Detection Kit I, BD Biosciences, Heidelberg, Germany). This method allows discrimination between early (annexin V+/PI-) and late (annexin V+/PI+) apoptotic, as well as necrotic (annexin V-/PI+) and viable (annexin V-/PI-) cells. The staining was performed according to the manufacturer’s instructions. Briefly, the cells were suspended in a buffer solution (10 mM Hepes/NaOH at pH 7.4, 140 mM NaCl, 2.5 mM CaCl_2_) at a final concentration of 1 × 10^6^ cells/ml. After addition of Annexin V-FITC, the cells were incubated for 15 min at room temperature without light exposure. Flow cytometry was performed using the FACS LSR II system (BD Biosciences). A total of 10,000 ungated events were acquired for each sample, and data were analysed with the BD FACS Diva® with the CSD module (BD Biosciences). In order to determine late apoptotic cells, PI was added to the samples. Flow cytometry was performed immediately thereafter.

### *In vivo* tumourigenicity study

A total of 300,000 T3M4 cells in 200 μl RPMI 1640 medium were injected subcutaneously behind the anterior forelimb of four-week-old athymic mice through a 26-gauge needle. The injection sites were examined daily for the appearance of tumours. Treatment was started on the seventh day after tumour inoculation. Mice were divided into groups receiving belinostat (0.1 mg/g, 5 × weekly), gemcitabine (0.15 mg/g, 2 × weekly), or a combination of both (Belinostat at 0.1 mg/g, 5 × weekly, plus gemcitabine at 0.15 mg/g, 2 × weekly) i.p., whereas the control group (sham) received only PBS (20 μl/g, 5 × weekly). Treatment was continued for 28 consecutive days, and tumours were measured twice weekly with Vernier callipers. Tumour volumes were calculated using the formula: tumour volume = (L × W²)/2, with L representing the length and W the width of the tumour [12].

The same treatment was performed after tumour inoculation by direct injection in the pancreatic tail via laparotomy. In this case, the tumours were compared at the end of 28- days of treatment.

After completion of treatment, the animals were sacrificed, and tumours were excised, fixed, and embedded in paraffin. The numbers of mice in each treatment cohort was 6. All experiments on animals were approved in accordance with German law on the care and use of laboratory animals.

### Immunohistochemistry

Paraffin-embedded tissue sections (2–3 mm thick) were deparaffinised in xylene and rehydrated in progressively decreasing concentrations of ethanol. Thereafter, slides were placed in washing buffer. Antigen retrieval was carried out by microwaving the tissue sections in 10 mM citrate buffer for 10 min. Sections were then incubated first with normal goat serum (DAKO Corporation, Carpinteria, CA, USA) for 45 min to block non-specific-binding sites, and then with a mouse monoclonal Ki-67 antibody, diluted 1:5 (DAKO Corporation). Incubation was performed for 18 h at 4 °C. Slides were then rinsed in washing buffer and incubated with a biotinylated secondary goat anti-mouse antibody (DAKO Corporation) for 45 min at room temperature. The slides were then washed in washing buffer, and each section was exposed to 100 μl DAB-chromogen substrate mixture (DAKO Corporation), then counterstained with Mayer’s haematoxylin. The sections were washed again, dehydrated in increasing concentrations of ethanol, and mounted with xylene-based mounting medium. Every staining was controlled with a negative control. For semi-quantitative analysis, slides were scored in a blinded manner by two observers. Using 400-fold image magnification, the positive and negative stained cells were counted at three independent units area. Next, the percentage of the positive stained cells was calculated.

### Statistical analysis

Results are expressed as mean ± standard error of the mean (SEM). Analysis of variance (ANOVA) was used to show an overall difference between groups, the Student *t* test for pairwise comparison of normal distributed parameters, and the Mann–Whitney *U* test for parameters without normal distribution. Significance was defined as *p* < 0.05. Graphical presentations were performed using GraphPad Prism version 4.02 for Windows (GraphPad Software).

## Results

### Effects of belinostat *in vitro*

#### *Antiproliferative effect of belinostat on pancreatic cancer cells*

**Figure 1:**
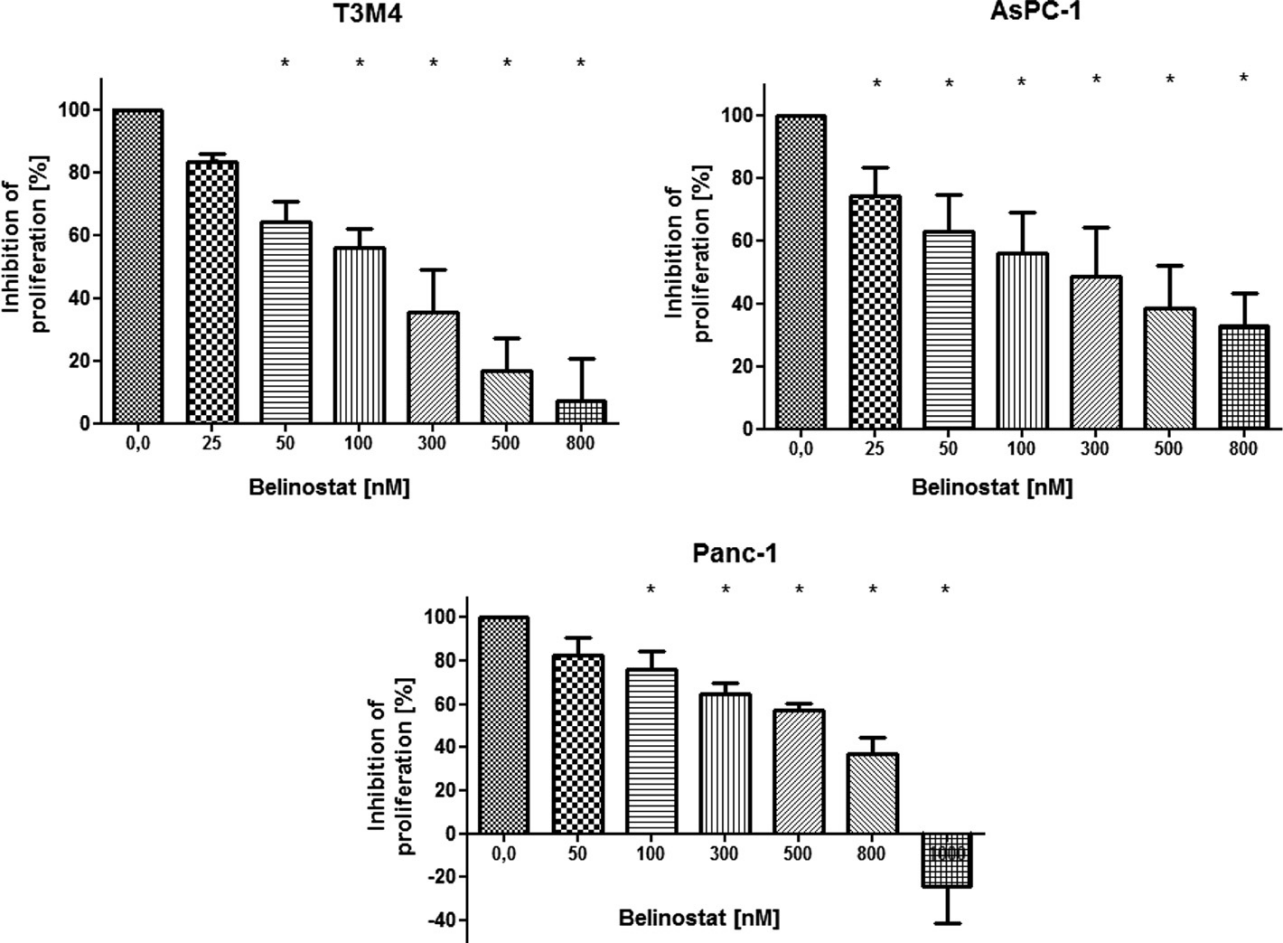
**Proliferation of PDAC cells (MTT assay).** Treatment with belinostat reduces the proliferation of pancreatic carcinoma cell lines *in vitro* in a dose-dependent manner. T3M4, AsPC-1 and Panc-1 cells were cultivated with increasing concentrations of belinostat. After 48 h, cell viability was determined. Data are presented as mean ± SEM. * *p* < 0.05 vs. control

Belinostat caused a significant dose-dependent decrease in cell proliferation in all cell lines tested. The ED50 concentrations for belinostat were ~100nM for T3M4, ~200nM for AsPC-1 and ~600 nM for Panc-1 (Figure 1).

#### *Apoptosis induction in pancreatic cancer cells by belinostat treatment*

**Figure 2:**
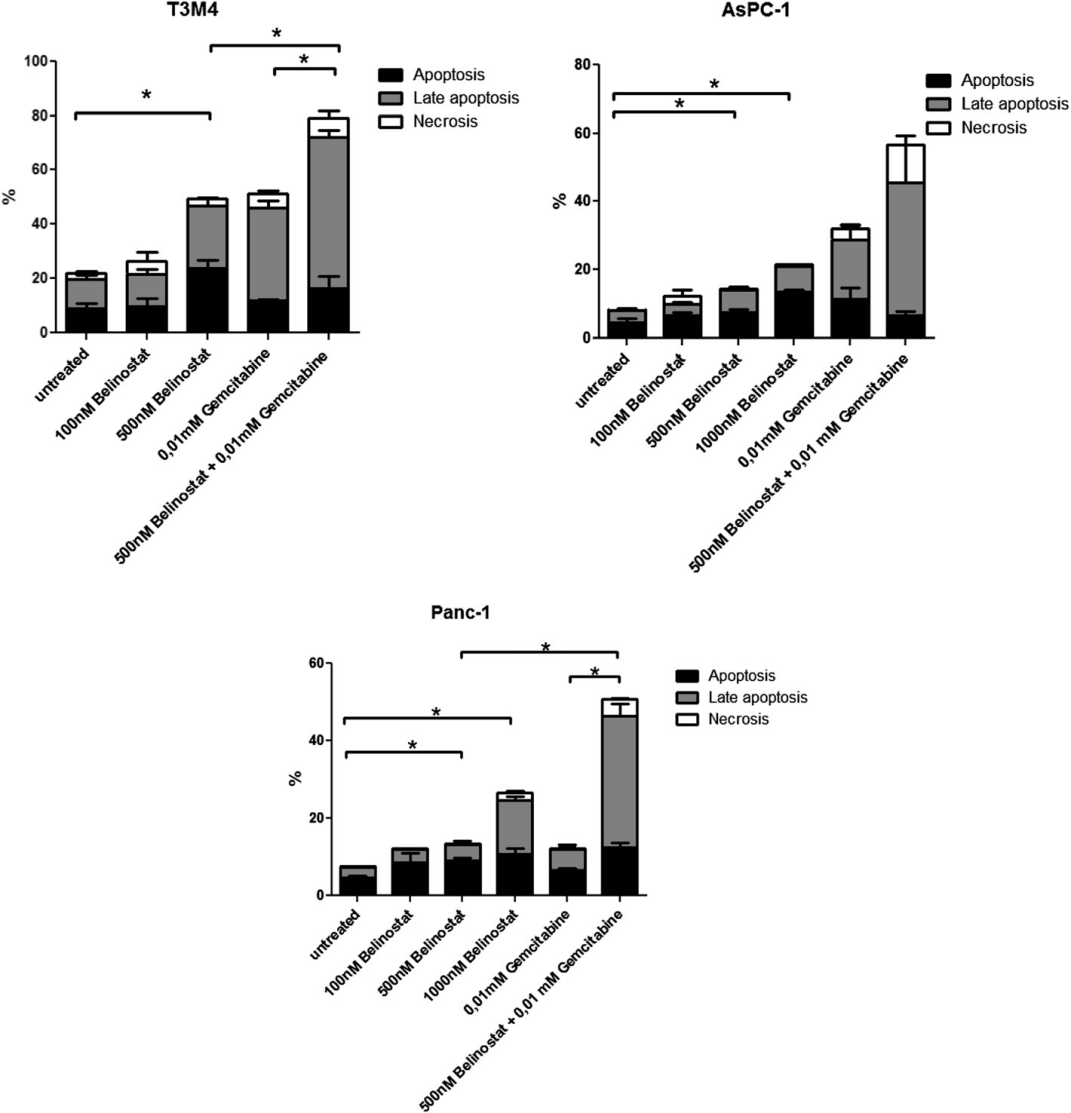
**Apoptosis and necrosis in PDAC cell lines after treatment with belinostat and (or) gemcitabine.** The percentage of dying tumour cells after treatment with belinostat, gemcitabine or belinostat + gemcitabine, stratified into early apoptotic, late apoptotic and necrotic fractions in the different PDAC cell lines. Belinostat increased apoptosis in a dose-dependent fashion in T3M4, AsPC-1 and Panc-1 cell lines. Moreover, the combination of belinostat and gemcitabine significantly enhanced apoptosis in T3M4 and Panc-1. Data are presented as mean ± SEM. * *p* < 0.05 vs. control

As shown in Figure 2, treatment with belinostat induced dose dependent apoptosis in all cell lines tested. The differences compared to control were significant at concentrations of 500 nM or more in all cell lines tested.

#### *Belinostat increases gemcitabine-mediated apoptosis in pancreatic tumour cells*

When concomitant use of both drugs (500 nM belinostat and 0,01 mM gemcitabine) was tested in T3M4, AsPC-1 and Panc-1 cells, the combined treatment significantly enhanced the proapoptotic activity compared to gemcitabine treatment alone in Panc-1 (~3 fold) and T3M4 (~1.5 fold) cells (Figure 2).

#### *Inhibition of histone deacetylation after belinostat treatment*

**Figure 3:**
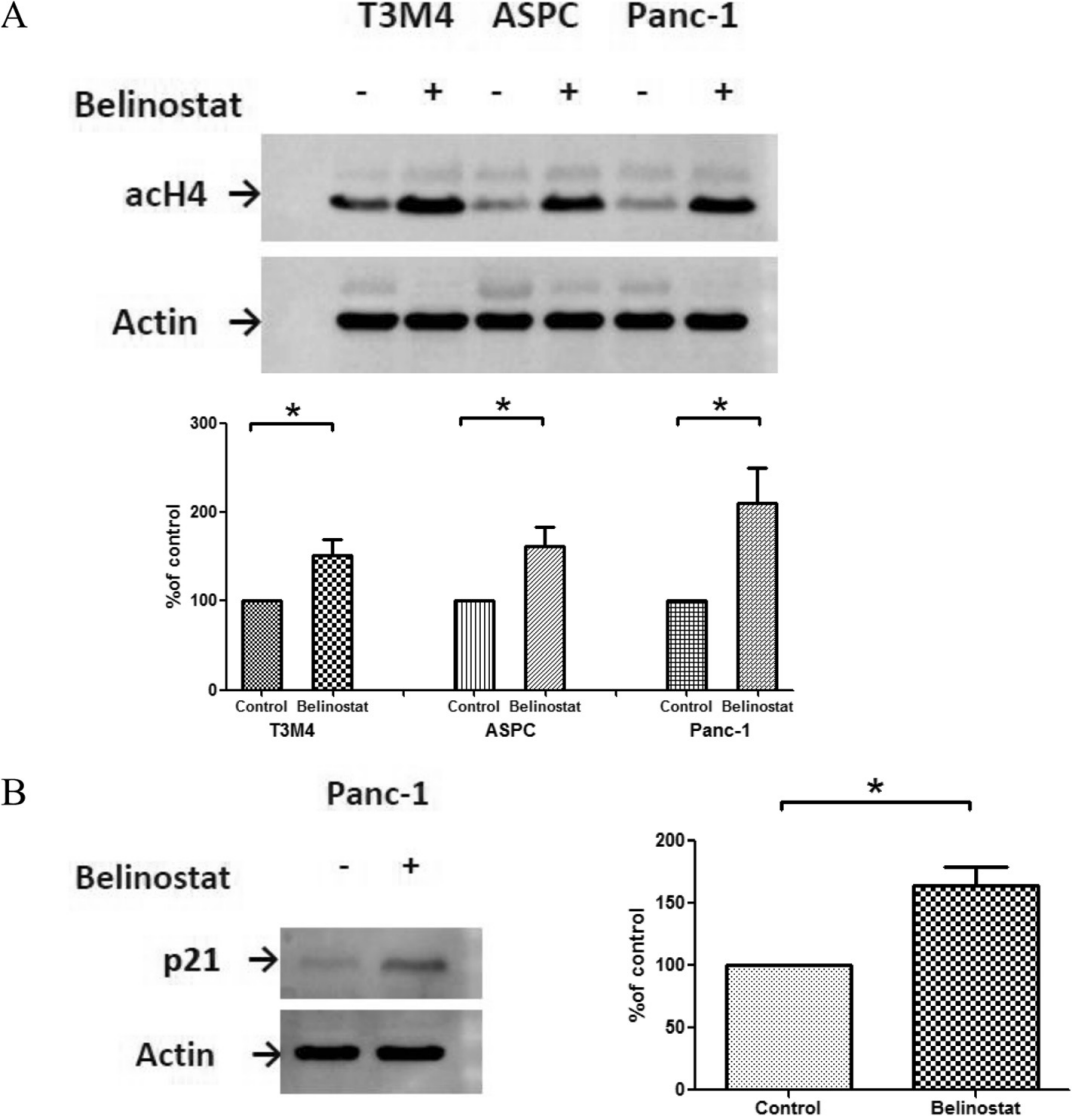
**Expression of acetylated histone H4 and p21**^**Cip1/Waf1**^**after belinostat treatment.** Representative Western blot analyses of H4 acetylation **(A)** and p21^Cip1/Waf1^ expression **(B)** after belinostat treatment (100nM for T3M4 and AsPC-1, 300nM for Panc-1). Equal loading of protein samples in each lane was confirmed using an anti-actin antibody. Densitometry shows increase of H4 acetylation (A) or p21^Cip1/Waf1^ expression (B) after the treatment of the tumour cells with belinostat (percentage of control). Data are presented as mean ± SEM. * *p* < 0.05 vs. control

In Western Blot analysis with an anti-ac-histone H4 antibody, treatment with belinostat significantly increased acetylation of histone 4 in all cell lines tested (Figure 3A).

#### *Belinostat induces expression of p21*^*Cip1/Waf1*^

In addition, belinostat was effective in increasing the level of p21^Cip1/Waf1^, which is related to HDACi-induced growth arrest in pancreatic carcinoma cells. Figure 3B demonstrates the increased expression of p21^Cip1/Waf1^ after belinostat treatment in Panc-1 cells.

### Inhibition of *in vivo* tumour growth by belinostat

**Figure 4:**
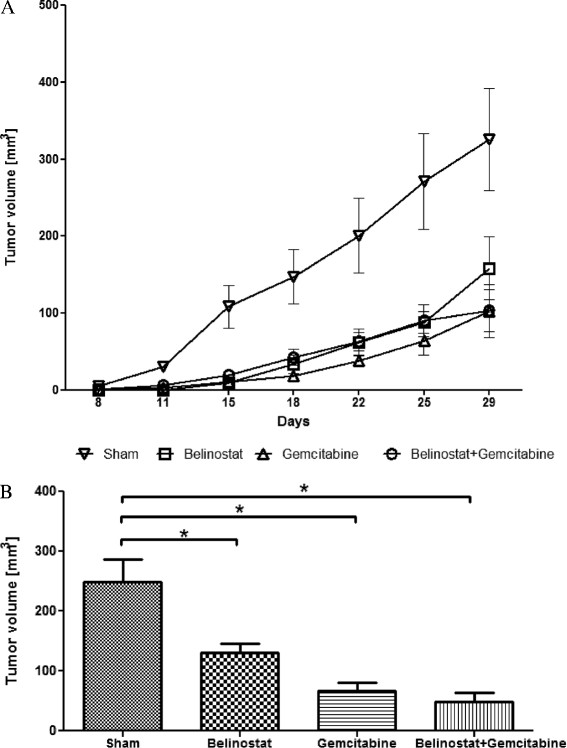
**Growth inhibition of xenograft tumours.** Subcutaneous **(A)** and intrapancreatic **(B)** xenograft tumour growth of T3M4 cells in nude mice treated with PBS (sham), belinostat, gemcitabine or belinostat + gemcitabine. The mean of the tumour volume was calculated from measurable tumours at each time point. In summary, tumours in the treatment groups were smaller compared to sham. The differences between the treatment groups were not significant. Data are presented as mean ± SEM. * *p* < 0.05

Tumours in the belinostat treatment group showed significantly reduced growth in both subcutaneous and intrapancreatic tumours compared with the control group, in *in vivo* experiments (Figures 4A and B). The combination of belinostat and gemcitabine therapy showed no additional growth inhibition.

**Figure 5:**
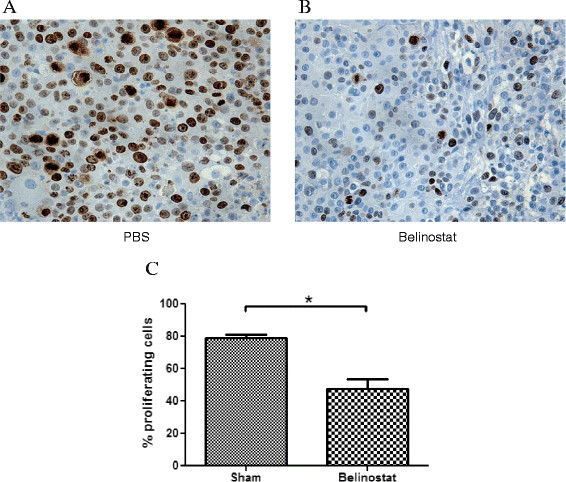
**Immunohistochemical analysis of cell proliferation in xenograft tumours.** Proliferating cells in sham **(A)** and belinostat **(B)** groups as assessed by an anti-Ki-67 antibody. The pictures were taken at 400-fold magnification. **(C)** Treatment with Belinostat led to a significant decrease in proliferating cells. Data (% proliferating cells per unit area) are presented as mean ± SEM. * *p* < 0.05

Routine hematoxylin/eosin histological examination showed no morphological differences between the tumours in the treatment and control groups. However, analysis of their proliferation rates (Figure 5) using an anti-Ki-67 antibody, showed a significantly lower number of proliferating cells per unit area in the belinostat group (47.8 ± 5.7 cells per high-power field) compared with the control group (79.1 ± 2.2).

## Discussion

PDAC remains a therapeutic challenge with a poor overall prognosis. Only surgery with adjuvant chemotherapy can achieve a long-term perspective in patients with localized tumours [13-15]. Adjuvant postoperative chemotherapy based on 5-FU or gemcitabine has been shown to improve the survival of these patients [16]. However, even under optimal treatment conditions, the 5-year survival rate doesn’t exceed 25% [13]. Additionally, palliative treatment in advanced tumour stages is associated with a poor prognosis and a median survival of about 6 months. To improve this situation, investigation of new therapeutic agents for PDAC treatment is essential.

The family of HDACi represents a novel approach in oncological research. In defined - predominantly haematological - tumour entities, HDACi have already passed the stage of experimental research and been investigated clinically [17]. Regarding PDAC, promising results have been shown using SAHA, TSA, butyrate and some other histone deacetylase inhibitors in experimental studies [6,18-20]. Belinostat is a novel member of the family with a distinct pan-HDAC inhibitory effect. It has been shown to be strongly effective in experimental settings of ovarian, bladder and colon cancer, as well as haematological tumour entities [9,21-24]. Consecutive clinical trials have proven an anti-tumour effect of belinostat as a monotherapy in T-cell lymphomas and thymomas. In addition, belinostat has demonstrated beneficial effects in combination with other anti-cancer drugs for the treatment of ovarian and bladder cancer, CUP, multiple myeloma and acute myeloid leukaemia. Despite these findings, no data are available concerning belinostat in the context of PDAC treatment. Consequently, in the present study, the efficacy of belinostat for PDAC treatment was investigated in experimental *in vitro* and *in vivo* settings for the first time.

Comparable to the results of previous studies in bladder [8], colorectal [25] or hepatocellular carcinoma [21], we found a strong dose dependent antiproliferative activity of belinostat in three pancreatic cancer cell lines (T3M4, Panc-1 and AsPC-1) with an IC50 concentration in the nanomolar range, similar to other tumour entities [8,25].

This antiproliferative effect can be explained by a strong proapoptotic activity in pancreatic cancer cells, demonstrated by annexinV/propidium iodide staining. This is in line with other studies on AML- [26] and hepatocellular carcinoma cells [21], underlining that apoptosis induction is an important mechanism of the anti-tumourous effect of HDACi, and particularly belinostat. As apoptosis induction is an important mechanism of anti-cancer chemotherapy [27], we tested the influence of concomitant use of belinostat and gemcitabine. As described in studies with other HDACi like trichostatin A [6] and 4-phenylbutyrate [18], the combination of gemcitabine and belinostat strongly enhanced the proapoptotic effects of each substance alone. This may be due to the expression of proapoptotic proteins like Caspase-8 and Bid, and activation of the gemcitabine-mediated JNK-pathway [18].

Increase in histone H4 acetylation has been shown to be helpful in monitoring belinostat activity [28]. Consequently, we examined belinostat-dependent expression of acH4 in PDAC cells. Acetylation of H4 was increased in all cell lines tested, confirming the inhibitory effect of belinostat on HDAC activity in pancreatic cancer cells.

Cyclin-dependent kinase inhibitor p21^Cip1/Waf1^ is a key protein participating in cell cycle regulation. Previous studies have shown that HDACi activates expression of p21^Cip1/Waf1^ through enhanced histone acetylation around the p21^Cip1/Waf1^ promoter [29]. We performed western blot analysis with treated and control Panc-1 cells to clarify the effect of belinostat on p21^Cip1/Waf1^ expression. Belinostat induced an upregulation of p21^Cip1/Waf1^, as has been described for other HDAC inhibitors in pancreatic cancer [18,30]. Increased expression of p21^Cip1/Waf1^ in these studies was associated with normalization of the cell cycle and induction of apoptosis.

Regarding the effect of belinostat *in vivo*, we observed that belinostat was an effective growth inhibitor of T3M4 pancreatic cancer cells in a nude mouse model. Mice treated with belinostat showed xenograft growth inhibition for more than 28 days after tumour inoculation, without any signs of toxicity. The reduction in the tumour volume was associated with decreased cell proliferation, as shown by Ki-67 immunohistochemistry. Similar observations in *in vivo* tumour models were shown in previous studies, e.g. in human ovarian cancer s.c. xenografts; the efficacy of the treatment with belinostat was further enhanced when a combination therapy with carboplatin was added [24]. Plumb et al. [22] described a significant dose-dependent growth delay of human colon tumour xenografts in mice after belinostat treatment, without signs of toxicity.

In contrast to our *in vitro* observations, we could not find an additional effect of combined therapy with belinostat and gemcitabine *in vivo*. A possible explanation for this discrepancy is the relatively high dosage of gemcitabine administered in the *in vivo* study. This might have covered a possible additional belinostat effect.

## Conclusion

In summary, this preclinical study using *in vitro* and *in vivo* pancreatic cancer models shows that belinostat is effective as a monotherapy of pancreatic cancer, primarily by inhibition of proliferation and induction of apoptosis. *In vitro* results revealed that belinostat can be successfully combined with gemcitabine to potentiate induction of apoptosis in the tumour cells. These findings should be confirmed in the clinical setting in PDAC patients.

## Abbreviations

HDAC: Histone deacetylase; HDACi: Histone deacetylase inhibitor; PDAC: Pancreatic ductal adenocarcinoma.

## Competing interests

The authors declare that they have no competing interests.

## Authors’ contributions

DD conceived the study, participated in its design and coordination, and drafted the manuscript; SA carried out *in vitro* and *in vivo* studies with cell culture and performed immunohistochemistry; TH participated in the study design and helped to draft the manuscript; IO performed immunoblot analysis; OW, KF and NG participated in the design of the study and revised the manuscript; JW participated in the overall design and study coordination, and finalized the draft of the manuscript. All authors read and approved the final manuscript.

## Pre-publication history

The pre-publication history for this paper can be accessed here:

http://www.biomedcentral.com/1471-2407/12/226/prepub

## References

[B1] Jemal A Siegel R Xu J Ward E Cancer statistics CA Cancer J Clin 2010 60 5 277 300 10.3322/caac.20073 20610543

[B2] Li D Xie K Wolff R Abbruzzese JL Pancreatic cancer Lancet 2004 363 9414 1049 1057 10.1016/S0140-6736(04)15841-8 15051286

[B3] Lockhart AC Rothenberg ML Berlin JD Treatment for pancreatic cancer: current therapy and continued progress Gastroenterology 2005 128 6 1642 1654 10.1053/j.gastro.2005.03.039 15887156

[B4] Welsch T Kleeff J Friess H Molecular pathogenesis of pancreatic cancer: advances and challenges Curr Mol Med 2007 7 5 504 521 17691965 10.2174/156652407781387082

[B5] Bolden JE JPM JR W Anticancer activities of histone deacetylase inhibitors NATURE REVIEWS 2006 5 769 784 10.1038/nrd2133 16955068

[B6] Donadelli M Costanzo C Beghelli S Scupoli MT Dandrea M Bonora A Piacentini P Budillon A Caraglia M Scarpa A Synergistic inhibition of pancreatic adenocarcinoma cell growth by trichostatin A and gemcitabine Biochim Biophys Acta 2007 1773 7 1095 1106 10.1016/j.bbamcr.2007.05.002 17555830

[B7] Kouraklis G Theocharis S Histone deacetylase inhibitors: a novel target of anticancer therapy (review) Oncol Rep 2006 15 2 489 494 16391874

[B8] Buckley MT Yoon J Yee H Chiriboga L Liebes L Ara G Qian X Bajorin DF Sun TT Wu XR The histone deacetylase inhibitor belinostat (PXD101) suppresses bladder cancer cell growth in vitro and in vivo J Transl Med 2007 5 49 10.1186/1479-5876-5-49 17935615 PMC2100044

[B9] Tumber A Collins LS Petersen KD Thougaard A Christiansen SJ Dejligbjerg M Jensen PB Sehested M Ritchie JW The histone deacetylase inhibitor PXD101 synergises with 5-fluorouracil to inhibit colon cancer cell growth in vitro and in vivo Cancer Chemother Pharmacol 2007 60 2 275 283 10.1007/s00280-006-0374-7 17124594

[B10] Giaccone G Rajan A Berman A Kelly RJ Szabo E Lopez-Chavez A Trepel J Lee MJ Cao L Espinoza-Delgado I Phase II study of belinostat in patients with recurrent or refractory advanced thymic epithelial tumors J Clin Oncol 2011 29 15 2052 2059 10.1200/JCO.2010.32.4467 21502553 PMC3107761

[B11] Gimsing P Hansen M Knudsen LM Knoblauch P Christensen IJ Ooi CE Buhl-Jensen P A phase I clinical trial of the histone deacetylase inhibitor belinostat in patients with advanced hematological neoplasia Eur J Haematol 2008 81 3 170 176 10.1111/j.1600-0609.2008.01102.x 18510700

[B12] Sanchez Y el-Naggar A Pathak S Killary AM A tumor suppressor locus within 3p14-p12 mediates rapid cell death of renal cell carcinoma in vivo Proc Natl Acad Sci U S A 1994 91 8 3383 3387 10.1073/pnas.91.8.3383 8159756 PMC43581

[B13] Neoptolemos JP Stocken DD Bassi C Ghaneh P Cunningham D Goldstein D Padbury R Moore MJ Gallinger S Mariette C Adjuvant chemotherapy with fluorouracil plus folinic acid vs gemcitabine following pancreatic cancer resection: a randomized controlled trial Jama 2010 304 10 1073 1081 10.1001/jama.2010.1275 20823433

[B14] Neoptolemos JP Stocken DD Tudur Smith C Bassi C Ghaneh P Owen E Moore M Padbury R Doi R Smith D Adjuvant 5-fluorouracil and folinic acid vs observation for pancreatic cancer: composite data from the ESPAC-1 and −3(v1) trials British journal of cancer 2009 100 2 246 250 10.1038/sj.bjc.6604838 19127260 PMC2625958

[B15] Oettle H Post S Neuhaus P Gellert K Langrehr J Ridwelski K Schramm H Fahlke J Zuelke C Burkart C Adjuvant chemotherapy with gemcitabine vs observation in patients undergoing curative-intent resection of pancreatic cancer: a randomized controlled trial Jama 2007 297 3 267 277 10.1001/jama.297.3.267 17227978

[B16] Cunningham D Chau I Stocken DD Valle JW Smith D Steward W Harper PG Dunn J Tudur-Smith C West J Phase III randomized comparison of gemcitabine versus gemcitabine plus capecitabine in patients with advanced pancreatic cancer J Clin Oncol 2009 27 33 5513 5518 10.1200/JCO.2009.24.2446 19858379

[B17] Bolden JE Peart MJ Johnstone RW Anticancer activities of histone deacetylase inhibitors Nat Rev Drug Discov 2006 5 9 769 784 10.1038/nrd2133 16955068

[B18] Ammerpohl O Trauzold A Schniewind B Griep U Pilarsky C Grutzmann R Saeger HD Janssen O Sipos B Kloppel G Complementary effects of HDAC inhibitor 4-PB on gap junction communication and cellular export mechanisms support restoration of chemosensitivity of PDAC cells Br J Cancer 2007 96 1 73 81 10.1038/sj.bjc.6603511 17164759 PMC2360208

[B19] Haefner M Bluethner T Niederhagen M Moebius C Wittekind C Mossner J Caca K Wiedmann M Experimental treatment of pancreatic cancer with two novel histone deacetylase inhibitors World J Gastroenterol 2008 14 23 3681 3692 10.3748/wjg.14.3681 18595135 PMC2719231

[B20] Kumagai T Wakimoto N Yin D Gery S Kawamata N Takai N Komatsu N Chumakov A Imai Y Koeffler HP Histone deacetylase inhibitor, suberoylanilide hydroxamic acid (Vorinostat, SAHA) profoundly inhibits the growth of human pancreatic cancer cells Int J Cancer 2007 121 3 656 665 10.1002/ijc.22558 17417771

[B21] Ma BB Sung F Tao Q Poon FF Lui VW Yeo W Chan SL Chan AT The preclinical activity of the histone deacetylase inhibitor PXD101 (belinostat) in hepatocellular carcinoma cell lines Invest New Drugs 2008 28 2 107 114 19172229 10.1007/s10637-009-9219-7

[B22] Plumb JA Finn PW Williams RJ Bandara MJ Romero MR Watkins CJ La Thangue NB Brown R Pharmacodynamic response and inhibition of growth of human tumor xenografts by the novel histone deacetylase inhibitor PXD101 Mol Cancer Ther 2003 2 8 721 728 12939461

[B23] Qian X Ara G Mills E LaRochelle WJ Lichenstein HS Jeffers M Activity of the histone deacetylase inhibitor belinostat (PXD101) in preclinical models of prostate cancer Int J Cancer 2008 122 6 1400 1410 18027850 10.1002/ijc.23243

[B24] Qian X LaRochelle WJ Ara G Wu F Petersen KD Thougaard A Sehested M Lichenstein HS Jeffers M Activity of PXD101, a histone deacetylase inhibitor, in preclinical ovarian cancer studies Mol Cancer Ther 2006 5 8 2086 2095 10.1158/1535-7163.MCT-06-0111 16928830

[B25] Kim JC Kim DD Lee YM Kim TW Cho DH Kim MB Ro SG Kim SY Kim YS Lee JS Evaluation of novel histone deacetylase inhibitors as therapeutic agents for colorectal adenocarcinomas compared to established regimens with the histoculture drug response assay Int J Colorectal Dis 2009 24 2 209 218 10.1007/s00384-008-0590-1 18830613

[B26] Stapnes C Ryningen A Hatfield K Oyan AM Eide GE Corbascio M Kalland KH Gjertsen BT Bruserud O Functional characteristics and gene expression profiles of primary acute myeloid leukaemia cells identify patient subgroups that differ in susceptibility to histone deacetylase inhibitors Int J Oncol 2007 31 6 1529 1538 17982680

[B27] Bold RJ Chandra J McConkey DJ Gemcitabine-induced programmed cell death (apoptosis) of human pancreatic carcinoma is determined by Bcl-2 content Ann Surg Oncol 1999 6 3 279 285 10.1007/s10434-999-0279-x 10340887

[B28] Marquard L Petersen KD Persson M Hoff KD Jensen PB Sehested M Monitoring the effect of belinostat in solid tumors by H4 acetylation APMIS 2008 116 5 382 392 10.1111/j.1600-0463.2008.00957.x 18452428 PMC2774150

[B29] Ocker M Schneider-Stock R Histone deacetylase inhibitors: signalling towards p21cip1/waf1 Int J Biochem Cell Biol 2007 39 7–8 1367 1374 17412634 10.1016/j.biocel.2007.03.001

[B30] Arnold NB Arkus N Gunn J Korc M The histone deacetylase inhibitor suberoylanilide hydroxamic acid induces growth inhibition and enhances gemcitabine-induced cell death in pancreatic cancer Clin Cancer Res 2007 13 1 18 26 10.1158/1078-0432.CCR-06-0914 17200334

